# A sustainable bilingual learning ecosystem in higher education: teacher and peer support under SDG 4.7

**DOI:** 10.3389/fpsyg.2026.1809800

**Published:** 2026-05-14

**Authors:** Fengqin Jin, Fengxiang Zhang

**Affiliations:** 1 Department of Early Childhood Education, Daegu University, Gyeongsan, Gyeongbuk, Republic of Korea; 2 College of Foreign Languages, Hebei University of Economics and Business, Shijiazhuang, Hebei, China

**Keywords:** Education for Sustainable Development (ESD), higher education, peer support, sustainable bilingual competence, Sustainable Development Goal 4.7 (SDG4.7), teacher support

## Abstract

**Purpose:**

Grounded in the theoretical framework of Education for Sustainable Development (ESD) and aligned with Sustainable Development Goal 4.7 (SDG 4.7), this study aims to explore how teacher support and peer support jointly contribute to the construction of a sustainable bilingual learning ecosystem in higher education. The study further examines the forms through which these two types of social support are provided and observes their associations with learners' motivation, self-efficacy, and psychological wellbeing, thereby offering theoretical support for the cultivation of sustainable bilingual competence.

**Methods:**

A qualitative research design was adopted. Semi-structured interviews were conducted with language-major teachers (*N* = 10) and undergraduate students (*N* = 10) from two comprehensive universities in Gyeongsangbuk-do, South Korea. Guided by the principles of constructivist grounded theory, the interview data were systematically analyzed using open coding, axial coding, and selective coding (three-level coding).

**Findings:**

The findings identify three core characteristics of a sustainable bilingual learning ecosystem: (1) teacher support operates through a dual-mode mechanism, encompassing structured linguistic empowerment, contextualized emotional care, and intercultural support; (2) peer support functions through synergistic effects, including cognitive collaboration and emotional resonance; and (3) Students internalize external support by developing intercultural psychological safety, thereby strengthening an interest-self-efficacy reinforcement cycle, ultimately achieving flow experiences and deep engagement in bilingual practices.

**Conclusion:**

Sustainable bilingual competence is cultivated within an interactive learning ecosystem, in which teacher support and peer support serve as key external social resources. Learners internalize these supports through interrelated psychological processes-such as psychological safety, motivation, and self-efficacy-thereby facilitating sustained engagement and development in bilingual learning contexts.

## 1 Introduction

Grounded in the framework of the United Nations Educational, Scientific and Cultural Organization (UNESCO) SDG 4.7, higher education is regarded as a critical stage for cultivating learners' capacities for sustainable development, including cross-contextual sustainable competencies such as cultural understanding, collaborative cooperation, and emotional regulation (Vare and Scott, 2007; UNESCO, 2020). In recent years, research on Education for Sustainable Development (ESD) has shifted its focus from an exclusive emphasis on curriculum content toward greater attention to how social interactions within learning environments support the continuous development of individual capacities (Kioupi and Voulvoulis, 2019). Particularly in higher education, learners are required to develop transferable competencies within complex, diverse, and constantly changing social contexts, rendering social interaction not merely a pedagogical tool but a core condition for the formation of sustainable competencies (Campbell et al., 2025). Bilingual education, due to its inherent features of intercultural interaction and emotional engagement, provides an important basis for both practicing and examining the principles of sustainable learning. From an ESD perspective, bilingual learning involves not only the acquisition of linguistic skills but also learners' ability to maintain learning motivation in challenging communicative environments. Such sustainable engagement is conceptualized as a learning ecosystem composed of external social support and internal psychological resources (Wu and Cai, 2025). Within this ecosystem, teacher support and peer support constitute key external social resources, while learning motivation, self-efficacy, and psychological wellbeing represent the core internal psychological processes and outcomes that sustain bilingual practice (Shen et al., 2024). Although existing studies have confirmed a positive association between social support and favorable learning outcomes (Shao et al., 2025), most of this research relies on cross-sectional quantitative designs that conceptualize support and psychological variables as static characteristics. As a result, these studies have not sufficiently captured how teacher support and peer support are provided and perceived in everyday instructional practices, nor how they are interconnected with learners' learning motivation, self-efficacy, and psychological wellbeing to jointly shape a sustainable bilingual learning ecosystem. Moreover, although these psychological processes are conceptually interrelated, teacher support and peer support often exert differentiated and pathway-specific influences in actual teaching practices. Distinguishing these forms of support analytically is therefore necessary to more clearly elucidate their respective mechanisms of action. To address these research gaps, the present study adopts a qualitative research design. Semi-structured interviews were conducted with 10 bilingual teachers and 10 undergraduate students majoring in bilingual-related programs at Korean universities. The study aims to understand how social support in bilingual classrooms is provided and perceived within authentic instructional contexts, and how such support is associated with students' learning motivation, self-efficacy, and psychological wellbeing. In doing so, the study seeks to clarify how a sustainable bilingual learning ecosystem is constructed and maintained, and how it supports the development of sustainable bilingual competence. Against this background, the present research attempts to explore how participants experience and articulate the interrelationships between support systems and psychological processes in real classroom contexts. The specific research questions are as follows:

RQ1: through what specific practices is teacher support manifested in bilingual classrooms, and how do these practices influence students' learning motivation and self-efficacy?

RQ2: as a social resource, how does peer support enhance students' psychological wellbeing, thereby supporting their engagement in bilingual learning?

RQ3: how are teacher support and peer support synergistically interconnected, and how do they interact with students' learning motivation, self-efficacy, and psychological wellbeing to jointly construct developmental pathways for sustainable bilingual learning?

By developing an ESD-oriented bilingual learning ecosystem model centered on social support and psychological processes, this study systematically explicates the internal mechanisms through which sustainable bilingual competence is formed and sustained. The findings are expected to provide both theoretical and empirical references for the implementation of ESD principles in higher education, as well as for the design and enactment of bilingual pedagogical practices that support students' psychological wellbeing and academic development.

## 2 Theoretical framework

### 2.1 ESD and sustainable bilingual competence

Within the framework of SDG 4.7, higher education is regarded as a critical stage for cultivating learners' capacities for sustainable development. Its goals extend beyond the transmission of knowledge to the development of sustainable competencies, including learning engagement, intercultural communication, critical reflection, and emotional regulation (UNESCO, 2020). Cristóvão et al. (2023) and Núñez et al. (2024) emphasize that, compared with specific curriculum content, the quality of social interactions, collaborative relationships, and emotional regulation within learning environments constitutes the core elements that determine whether sustainable competencies can be continuously developed. Bilingual education, due to its inherent characteristics of intercultural interaction, emotional engagement, and ongoing processes of meaning negotiation, is regarded as an educational approach that is particularly conducive to supporting the development of sustainable competencies. Bilingual education enables learners to acquire intercultural expressive and interpretive abilities, problem-solving skills in complex contexts, as well as emotional regulation and psychological resilience when confronting communicative challenges (Kioupi and Voulvoulis, 2019; Jin and Ye, 2022; Qian et al., 2022). These competencies are explicitly defined as core competencies within the ESD framework (UNESCO, 2024). Furthermore, the interactive nature of bilingual learning and its associated processes of identity construction make it an important context for examining the formation of “sustainable learning ecosystems” and “sustainable bilingual competence” (Blašková et al., 2021). Accordingly, from an ESD perspective, the focus of bilingual education should shift from the mere acquisition of linguistic skills to the sustained development of bilingual competence within a learning ecosystem. This perspective underscores the need to examine how teacher support and peer support are jointly interconnected in maintaining and enhancing learners' learning motivation, self-efficacy, and psychological wellbeing.

### 2.2 Sustainable bilingual competence: a dynamic capability perspective

Sustainable bilingual competence can be understood through the theoretical lens of dynamic capability. From this perspective, competence is not conceived as a static individual trait but as a dynamic construct that is continuously formed and developed through ongoing interactions between learners and supportive environments (Amoozegar et al., 2025). Within the ESD framework, bilingual competence is regarded as a form of sustainable dynamic capability, characterized by the following features: (1) learning motivation is closely associated with learners' willingness to invest effort in bilingual engagement (Ryan and Deci, 2020); (2) self-efficacy serves as a key psychological resource that enables learners to persist in bilingual practice when confronted with communicative setbacks (Bandura et al., 1999; Qian et al., 2022); and (3) teacher support, peer collaboration, and emotional regulation are jointly associated with the quality of bilingual thinking and expression, as well as learners' psychological experiences (Thomsen et al., 2025; Green et al., 2022). Accordingly, from an integrative perspective that combines dynamic capability theory with ESD, sustainable bilingual competence should not be viewed as a fixed endpoint defined by a particular level of language proficiency. Rather, it represents a form of developmental potential that learners construct within a supportive learning ecosystem through the continuous accumulation of learning interest, self-efficacy, and positive emotional experiences (Dong et al., 2021; López et al., 2024).

### 2.3 Self-determination theory, social cognitive theory, and sustainable learning motivation

Self-Determination Theory (SDT) provides a robust foundation for understanding the psychological pathways that support sustainable learning. When learning environments continuously satisfy students' basic psychological needs-autonomy (e.g., choice in learning), competence (e.g., effective learning scaffolds), and relatedness (e.g., positive teacher-student and peer relationships)-learning motivation becomes internalized from external sources, manifesting as enhanced learning interest, persistence, and psychological wellbeing (Ryan and Deci, 2020; Weng et al., 2025). In bilingual education, teacher-provided autonomy support, such as encouraging personalized expression in the target language, can strengthen students' learning interest and sense of ownership (Bonneville-Roussy and Evans, 2024). Competence support, such as structured feedback and scaffolding of instructional tasks, helps learners maintain self-efficacy and persistence when confronted with language-learning challenges (Wieser et al., 2024). Relatedness support, including an inclusive classroom climate and emotional care, effectively alleviates language anxiety, enhancing learners' psychological safety and wellbeing during classroom participation (Huawei and Jenatabadi, 2024). Social Cognitive Theory further emphasizes that self-efficacy is a critical mediator between social support and sustained learning behaviors. It directly influences learners' willingness to engage in challenging instructional tasks, their resilience in the face of difficulties, and their capacity for emotional regulation (Oh and Pyo, 2023). Within bilingual learning contexts, high self-efficacy not only predicts better immediate performance but also forecasts learners' future engagement in authentic language use (Qian et al., 2022). Moreover, learning motivation and self-efficacy are mutually reinforcing in a cyclical relationship: engagement driven by interest leads to successful experiences, which enhance self-efficacy, thereby fostering deeper and more sustained learning motivation. This cyclical process constitutes a positive feedback loop that is central to understanding the developmental pathways of sustainable bilingual competence (Street et al., 2022).

### 2.4 Teacher support, peer support, and the sustainable bilingual learning ecosystem

Social support within learning environments constitutes a critical external condition for the cultivation of sustainable competencies (Ben-Eliyahu, 2021; Bohm et al., 2024). In bilingual classrooms, teacher support and peer support serve as key social resources linking learning motivation, self-efficacy, psychological wellbeing, and learning engagement (Liu et al., 2021; Shi et al., 2025). This linkage can be conceptualized in three primary dimensions: (1) Teacher support: through instructional scaffolding, feedback guidance, emotional care, and cultural sensitivity, teachers enhance students' perceptions of competence and psychological safety (Thomsen et al., 2025). Such support facilitates learning engagement, reduces fear of communicative failure, and strengthens emotional regulation capabilities (Zhang et al., 2024); (2) Peer support: collaborative interactions among peers promote co-construction of meaning (Peralta Hernández and Tirado Segura, 2023), alleviate stress through emotional support, and reinforce affective bonds that sustain ongoing learning (Green et al., 2022; Sun et al., 2025). Peer collaboration contributes to the formation of a mutually supportive and sustainable bilingual learning community (Martín-García et al., 2020); and (3) Pathway to sustainable bilingual competence: existing studies suggest a potential pathway in which external social support enhances learning motivation and self-efficacy, which in turn strengthens psychological wellbeing, promotes sustained learning engagement, and ultimately supports the development of sustainable bilingual competence (Liu et al., 2021; Huang, 2025). However, current research lacks qualitative evidence that illuminates how these forms of support operate in daily bilingual teaching practice, how they are perceived by teachers and students, and how they interact with learners' internal psychological processes to collectively construct a sustainable learning ecosystem. Against this background, the present study employs a qualitative research design to investigate how interactions among teachers, peers, and students in bilingual classrooms converge into an ecosystem that supports the development of sustainable bilingual competence, thereby addressing this gap in the literature. The theoretical framework integrates multiple perspectives: the learning ecosystem perspective serves as the overarching analytical lens to examine how social support and individual psychological processes interrelate within a concrete context; Self-Determination Theory (SDT) explains how external support is internalized into learning motivation and psychological wellbeing through satisfaction of basic psychological needs; and Social Cognitive Theory (self-efficacy) elucidates how learners develop confidence and persist in engaging with challenging communicative tasks under supportive conditions. These theoretical perspectives are not intended to test isolated causal relationships but collectively provide an interpretive basis for deeply understanding participants' lived experiences in authentic bilingual classroom contexts.

## 3 Methods

### 3.1 Research design

To investigate how teacher support and peer support jointly contribute to the construction of a sustainable bilingual learning ecosystem, and to understand learners' meaning-making, emotional experiences, and collaborative processes within instructional interactions, this study employed a qualitative research design (Clifton and Grushka, 2022; Moar et al., 2024). The focus was on understanding and explicating concrete processes in teaching practice rather than examining linear causal relationships among variables. Some research on ESD similarly emphasizes the necessity of qualitative approaches to observe ecological interactions within learning environments, which better explain how sustainable competencies emerge in instructional contexts (Brandt et al., 2022; Munasi, 2024). This study is theoretically framed via the *sustainable learning ecosystem* perspective (Song, 2024), conceptualizing the bilingual learning process as a system shaped by social, emotional, and motivational factors. The study particularly examines how the interaction between teacher support and peer support relate to the development of learning motivation, self-efficacy, and psychological wellbeing, thereby supporting the formation of sustainable bilingual competence. This design aligns closely with ESD principles emphasizing both competence development and learning ecosystem dynamics (Holst, 2023). Moreover, this study adopts constructivist grounded theory to explore how social support operates within higher education bilingual classrooms, and how such support is experienced and interpreted by teachers and students. This approach is consistent with the ESD perspective that views bilingual learning as a socialized process rather than a series of quantifiable outcomes. The rationale for selecting constructivist grounded theory includes: (1) its emphasis on co-construction of meaning between researcher and participants, which aligns with the study's focus on the lived experiences and narratives of sustainable bilingual learning; (2) its provision of a systematic yet flexible analytic framework, allowing theory to emerge inductively from participants' narratives while engaging in dialogue with existing theoretical perspectives, such as SDT and Social Cognitive Theory. The purpose of this study is not to test pre-specified causal hypotheses but to construct an experience-based explanatory model elucidating how a sustainable bilingual learning ecosystem is constructed and maintained in authentic classroom practice. All supplementary materials, including the sampling framework, interview protocol, and coding procedures, are provided in the supplementary (Tables S1–S3).

### 3.2 Research participants

This study employed purposeful sampling (Dahal et al., 2024) to recruit participants who could provide in-depth descriptions of teaching practices, interaction mechanisms, and learning experiences. Ultimately, 10 language-major teachers and 10 undergraduate students from two comprehensive universities in Gyeongsangbuk-do, South Korea, were recruited (Table 1). In addition, the linguistic background of the student participants reflects a structured bilingual learning context. All students were enrolled in English-related majors, where English functioned as a primary academic language, while Chinese was learned as a second language within formal university coursework. Their bilingual experience was therefore characterized by the use of English for academic thinking and Chinese for developing communicative and intercultural competence. This configuration ensured that participants engaged in authentic bilingual tasks while still encountering varying levels of linguistic and intercultural challenge, which is central to examining how social support contributes to sustained engagement in bilingual learning. Adopting a dual perspective from both teachers and students enabled a more comprehensive construction of the *sustainable bilingual learning ecosystem* model and allowed for the analysis of how social support interacts across different thematic areas, thereby revealing pathways in the development of psychological processes. This research design aligns with the emphasis in sustainable education studies on learning communities and the importance of multi-stakeholder, multi-method observation (Vičič Krabonja et al., 2024).

**Table 1:** Demographic profile of teacher and student participants.

Participant group	*N*	Key characteristics
Teachers	10	Teaching experience: 5–12 years (mid-career to senior level)
		Academic background: english language and literature, applied linguistics, Teaching English to Speakers of Other Languages (TESOL), and related fields
		Teaching practice: teaching Chinese as a second language (CSL) within English-major programs
Students	10	Academic level: second- to fourth-year undergraduate students
		Major: english-related programs
		Bilingual proficiency: Chinese proficiency approximately equivalent to Hanyu Shuiping Kaoshi (HSK) Levels 3–5
		Perceived social support: varied levels of perceived classroom social support

### 3.3 Data collection

The primary data for this study were collected through semi-structured interviews. Previous research indicates that semi-structured interviews are particularly effective for capturing learners' emotional regulation, interests, and relational interactions, making them well-suited for understanding internal structures in sustainable learning (Chang et al., 2023; Katsantonis and McLellan, 2023). The interviews focused on the following thematic areas: (1) specific forms of teacher support, including instructional scaffolding, feedback, emotional care, and culturally sensitive support; (2) peer support, collaboration, and overall classroom climate; (3) development of students' learning motivation, self-efficacy, and willingness to sustained engagement; (4) learners' perceptions and understanding of the sustainability of the bilingual learning environment; (5) how bilingual competence is continuously developed during everyday classroom interactions. Each interview lasted approximately 40 min. With participants' consent, all interviews were audio-recorded and subsequently transcribed verbatim for analysis using *NVivo* 12. To protect participants' anonymity, teacher interviews were coded as T1-T10, and student interviews were coded as S1-S10. All original quotations presented in this study are referenced using these codes.

### 3.4 Data analysis

Data analysis is carried out under the procedures of constructivist grounded theory, drawing on a systematic coding framework while maintaining an interpretive and reflexive stance throughout. The analysis proceeded through three iterative stages: (1) open coding: interview transcripts were analyzed line by line to identify initial concepts related to teacher support, peer interaction, learning motivation, self-efficacy, psychological wellbeing, and sustained engagement in bilingual learning. This stage yielded a total of 118 initial codes; (2) axial coding: initial codes were compared, grouped, and integrated based on conceptual similarity and relational patterns. Through constant comparison of teacher and student narratives, the analysis focused on the conditions, interaction processes, and perceived outcomes of social support within the bilingual learning environment, further refining and consolidating higher-level categories; and (3) selective coding: the *sustainable bilingual learning ecosystem* was identified as the core category, integrating teacher support, peer support, and learners' internal psychological processes (motivation, self-efficacy, and psychological wellbeing). Relationships among categories were systematically examined to construct an explanatory model, illustrating how external social support is internalized and transformed into sustained bilingual learning engagement and competence development.

### 3.5 Research trustworthiness and rigor

To enhance the rigor and credibility of the study, multiple strategies aligned with qualitative research standards were employed: (1) coder consistency check: two researchers independently coded approximately 30% of the interview transcripts. They then engaged in iterative discussions to compare interpretations and resolve discrepancies, aiming to reach a shared understanding and thereby strengthen the stability of the analysis; (2) theoretical saturation assessment: the saturation of categories was evaluated through constant comparison of new data with existing categories. Data saturation was defined as the point at which no new conceptual properties, dimensions, or relationships emerged from the data, particularly in relation to teacher support, peer support, and internal psychological processes. During the later stages of data collection and analysis, no substantively new categories or relationships were identified, and subsequent interviews served to confirm the stability and completeness of the emerging analytical framework; (3) member checking: selected participants were invited to review the preliminary findings and interpretations. Specifically, participants were provided with summarized thematic interpretations and representative analytical claims rather than full interview transcripts. They were asked to evaluate whether these interpretations accurately reflected their experiences and whether any important meanings had been overlooked. Their feedback supported the credibility of the analytical interpretations and assisted in refining category descriptions, with minor clarifications incorporated into the final analysis; and (4) reflexive memos: throughout the research process, the researchers maintained reflexive memos documenting research assumptions, personal perspectives, and analytical decisions. This practice enhanced transparency and minimized unexamined bias, consistent with the emphasis on reflexivity in qualitative research (Lincoln and Guba, 1985; Enworo, 2023).

## 4 Findings

This study employed constructivist grounded theory and systematically analyzed interview transcripts from both teacher and student perspectives using three-level coding (open, axial, and selective coding). The aim was to explore how teacher support and peer support jointly construct a sustainable bilingual learning ecosystem in higher education bilingual classrooms. The analysis ultimately converged into three core categories: (1) structured practices of teacher support; (2) cognitive interaction and emotional resonance patterns of peer support; and (3) mechanisms through which students internalize external support into a “interest-self-efficacy-flow experience” cycle for sustainable bilingual competence development. An overview of these three core categories along with their subthemes and conceptual meanings is presented in Table 2.

**Table 2:** Overview of core themes in the sustainable bilingual learning ecosystem.

Core theme	Subtheme	Conceptual meaning
Dual-pathway model of teacher support	Structured linguistic empowerment	Teachers provide instructional scaffolding, task structuring, and strategy-focused guidance to support language development and the cultivation of communicative confidence
	Contextualized emotional and intercultural care	Teachers create psychologically safe learning environments by offering corrective feedback, explicating intercultural communicative pressures, and providing emotional support, thereby encouraging students to take communicative risks
Synergistic effects of peer support	Cognitive collaboration	Peer interactions generate diverse perspectives, feedback, and models, which stimulate language use and facilitate the co-construction of meaning
	Emotional resonance	Shared experiences and mutual encouragement help regulate emotions and sustain learning engagement
Internalization of support	Intercultural psychological safety	External emotional support is internalized as a sense of safety for engaging in communicative risk-taking in intercultural contexts
	Reinforced interest–self-efficacy cycle	Interest-driven engagement and successful learning experiences mutually reinforce one another, thereby strengthening learning motivation and self-efficacy
	Bilingual communication and identity investment	Deeply engaged bilingual interaction experiences contribute to the consolidation of long-term bilingual identity and promote sustained participation in bilingual practices

### 4.1 Theme one: dual-pathway model of teacher support

#### 4.1.1 Structured linguistic empowerment

In bilingual education, competence development depends not only on the transmission of linguistic knowledge but also on supportive strategies that allow students to gradually build communicative efficacy through “challenging but achievable” tasks. As T5 stated, “When teaching academic Chinese writing, I do not provide a template directly. I first guide students to analyze the structure of texts with different topics, then let them practice through imitation, and finally provide targeted corrective feedback. The goal is for students to understand how language serves communicative purposes, not merely to correct errors.” T1, T3, and T8 shared similar structured empowerment strategies, such as stepwise linguistic scaffolding and explicit instruction in communicative strategies, enabling students to develop language competence through mastery and adaptation. Students' accounts corroborated this empowerment pathway. S8 noted, “When I give classroom presentations, the teacher helps me organize the logical framework and tells me how to use linking words to make my arguments more coherent. This is not just about correcting language mistakes-it teaches me how to express my ideas more effectively in Chinese, which makes me more confident in my next presentation.” S2 and S5 described similar experiences, highlighting that teachers provided personalized support according to their individual proficiency levels, enabling the synchronous development of language skills and personal expression style.

#### 4.1.2 Contextualized emotional and intercultural care

In addition to structured competence development, creating a safe learning environment through emotional support and intercultural understanding is crucial for alleviating language anxiety and encouraging risk-taking. T2 stated, “When organizing intercultural role-plays, I discuss potential cultural misunderstandings in advance and emphasize that communicative intent is more important than linguistic perfection.” T4, T6, and T9 similarly emphasized attending to students' emotional states during second-language use, particularly when addressing culturally sensitive topics, providing acceptance and emotional support. Alongside skill empowerment, teachers also provided emotional care. T7 recounted, “A student was so anxious before the final presentation that she could not sleep, fearing her accent would be ridiculed. I told her: ‘Your prepared content is valuable; the audience wants your insights, not a broadcaster.' I practiced with her several times, focusing on content delivery rather than pronunciation. When she finally completed it successfully, all the pressure turned into a sense of achievement.” Students' experiences were imbued with emotional significance. S3 described, “I was very scared the first time I participated in a group debate in Chinese. But before I went on stage, the teacher patted my shoulder and said: ‘Your argument is excellent; it deserves to be expressed in any language.' This turned my fear of making mistakes into willingness to try.” S4, S9, and S10 similarly reported that the care and support provided by teachers during criticism, failure, or uncertain communicative situations helped them maintain psychological stability and learning resilience. Teachers' dual-pathway support not only facilitated students' development of communicative competence but was also interpreted in participants' narratives as creating a learning environment characterized by psychological safety, intercultural understanding, and emotional connection. This approach shifted bilingual learning from a “task performance” orientation toward a “development-oriented communicative competence” orientation.

### 4.2 Theme two: collaborative effects of peer support

#### 4.2.1 Cognitive collaboration

Peers hold an important role both cognitively and emotionally. S1 described the significance of peer feedback: “when preparing for the HSK speaking test, we formed a mock interview group. Peers would point out logical gaps in my answers and suggest more authentic ways of expressing myself. This feedback is not judgmental; it is a process of solving problems together.” S6 similarly emphasized, “Good peer collaboration is: I feel that if you use ‘fǎn'ér' here, the contrast becomes sharper, rather than simply saying ‘this is wrong.' They think together with me on how to optimize, which makes me feel I am not fighting alone.” S2, S3, and S8 also noted that constructive criticism, collaborative problem-solving, and facing challenges together form a stable cognitive support system, helping them overcome individual thinking limitations and access richer linguistic resources and strategies.

#### 4.2.2 Emotional resonance

S1, S2, S3, and S9 reported that peer support operates not only at the cognitive level but also at the emotional level. S9 summarized the power of collective belonging: “During the toughest period of exam preparation, a few of us formed a learning community. We stayed up late together researching, quizzed each other on vocabulary, shared anxieties, and celebrated every small progress. That sense of advancing together was the greatest motivation to persist.” S6 also emphasized, “When you get frustrated because you just cannot understand a grammar point, a classmate says: ‘I struggled the same way at first; I'll show you my clumsy method.' That sense of being understood immediately alleviates feelings of loneliness.” This emotional resonance, as described by participants, is regarded as an important social support resource within a sustainable learning ecosystem. It helps students cope with stress, anxiety, and isolation during long-term and uncertain bilingual learning processes. Through the dual synergy of cognition and emotion, peer support provides students with a sustainable social support network, enabling bilingual competence to continuously circulate, be maintained, and be strengthened within the learning community.

### 4.3 Theme three: internalization and transformation of support

#### 4.3.1 Establishing intercultural psychological safety

S1, S4, and S9 described that teachers' emotional and intercultural care combined with peers' emotional resonance collectively created the psychological precondition for “daring to take risks.” S3 emphasized, “The greatest barrier in bilingual communication is the fear of being judged. But when teachers and classmates show understanding and treat mistakes as part of the learning process, this fear becomes a manageable risk.” S7 added, “Knowing that even if I ‘mess up,' it will be seen as a valuable attempt rather than a failure makes me more willing to speak up.” Analysis indicates that this internalized psychological safety is not a peripheral condition in the sustainable bilingual learning ecosystem; rather, it repeatedly emerged in participants' narratives as an essential experiential structure supporting sustained engagement in bilingual learning. More importantly, this form of intercultural psychological safety can be understood as learners' perceived legitimacy to engage in meaning-making across linguistic and cultural boundaries, even when their expressions are partial or non-native-like. It thus operates as a mediating experiential condition through which communicative risk is reinterpreted as a manageable and developmentally meaningful challenge, enabling learners to move from hesitation to active participation.

#### 4.3.2 Strengthening the interest-self-efficacy reinforcement cycle

S5, S8, and S10 described that initial interest guides deeper exploration, and local successes achieved during exploration enhance communicative confidence (self-efficacy), which in turn strengthens or sustains interest. S8 described a complete pattern from interest to self-efficacy: “initially attracted by Chinese martial arts films, I spontaneously watched many original soundtracks and mimicked lines (interest driving exploration). The first time I roughly retold a film plot in class and received teacher affirmation (success/external recognition), I suddenly felt that I can actually say something interesting in Chinese (confidence/self-efficacy)! Now, not only am I interested in martial arts films, but I am also curious about analyzing cultural metaphors in Chinese and challenging myself with more complex film reviews (deeper interest and exploration).” S5 and S10 reported similar experiences. This recurring Interest-Self-Efficacy Reinforcement Cycle emerged in the data as a core intrinsic motivational mechanism, rather than a single or incidental learning event.

#### 4.3.3 Bilingual flow experience and identity

**Figure 1:**
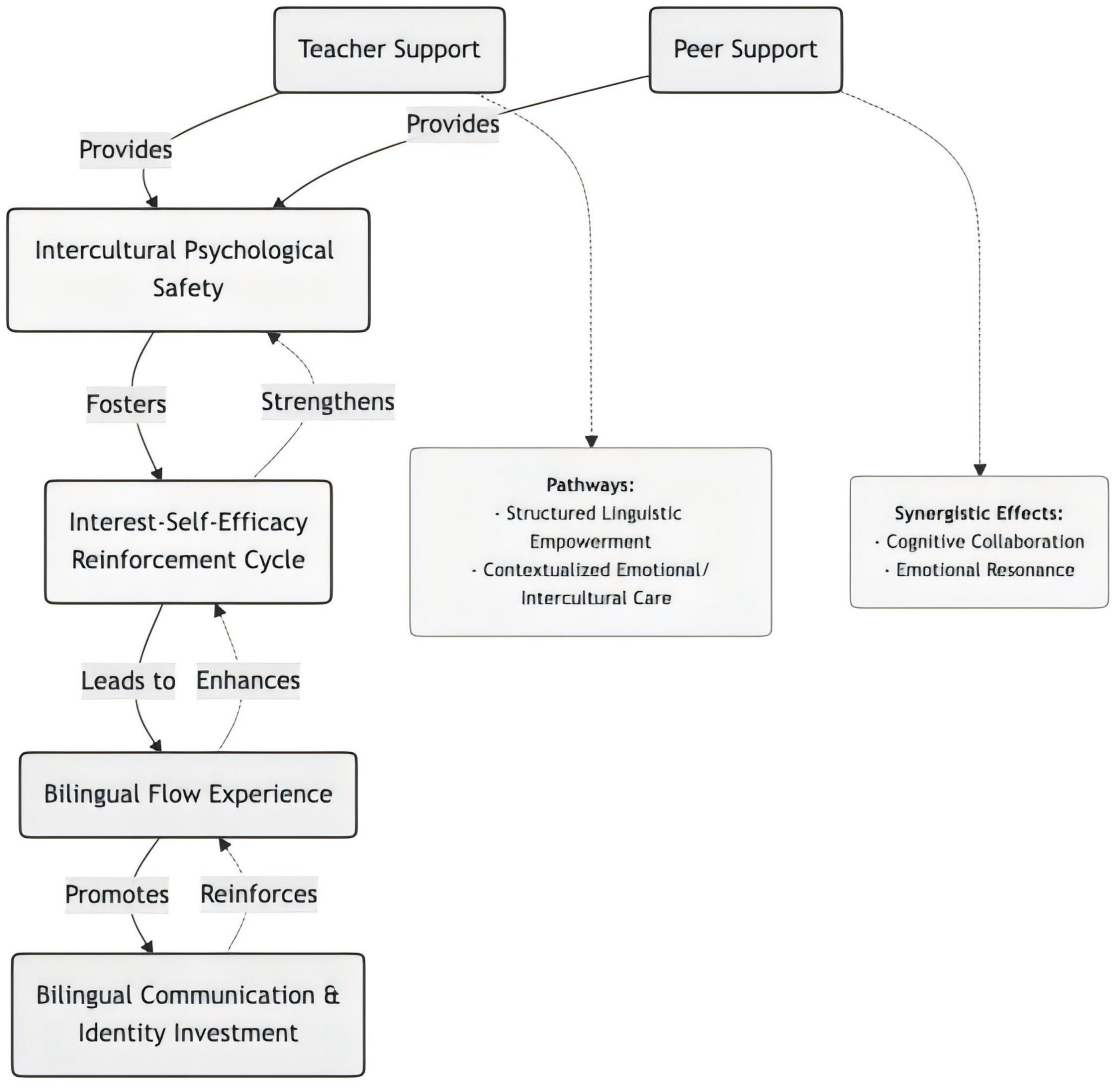
Model of the sustainable bilingual learning ecosystem.

When external support (teacher empowerment and peer collaboration) is fully internalized and connected with psychological safety and the interest-self-efficacy cycle, some students experience a highly immersive “flow” state in bilingual activities. S3 described, “When the group discussion topic is familiar to me and peers' encouragement helps me fully relax, I become completely immersed in communicating my ideas, forgetting which language I am using; my thinking and expression become exceptionally smooth.” S4 and S9 reported similar experiences, highlighting “challenge-skill balance” and “sense of autonomous control.” This flow experience has dual significance in participants' narratives: (1) it enhances engagement and enjoyment in individual bilingual interactions; and (2) it transforms into positive affective memory toward bilingual practice itself. As S9 noted, “After experiencing moments when ‘everything clicks,' even knowing difficulties remain, I still long to engage in that depth of interaction again.” Therefore, flow is understood in this study as a critical outcome of the interaction between external support and internal motivation in learners' experiential domain, rather than a predetermined developmental endpoint. Based on these three themes, this study proposes an explanatory model of the sustainable bilingual learning ecosystem (see Figure 1), summarizing how teacher support, peer support, and learners' psychological processes are interconnected in participants' experiences.

The model depicts the interaction between external social support (teacher and peer support) and internal psychological processes. External support fosters intercultural psychological safety, which activates the Interest-Self-Efficacy Reinforcement Cycle and facilitates flow experience in bilingual communication. These processes jointly sustain engagement and support long-term identity investment. The model represents a dynamic ecosystem with reciprocal pathways rather than a strictly linear sequence.

## 5 Discussion

### 5.1 Dual-pathway mechanism of teacher support

Teacher support in bilingual classrooms manifests as a dual-pathway mechanism-structured linguistic empowerment and contextualized emotional and intercultural care-operating in parallel and complementing each other. This not only directly addresses RQ1 but also illustrates how teacher support systematically cultivates sustainable bilingual competence by satisfying learners' basic psychological needs at multiple levels.

(1) Structured linguistic empowerment pathway precisely meets students' competence needs (Ryan and Deci, 2020). As T5 and T8 described, through scaffolding, task decomposition, and strategic feedback (rather than simple error correction), teachers transform complex bilingual communicative tasks into a series of challenging yet achievable steps. S8 and S2's experiences confirmed that such support enabled them not only to master linguistic forms but also to understand how language serves communicative purposes, thereby establishing a solid sense of communicative efficacy. This efficacy is a key predictor, according to social cognitive theory, of whether individuals persist when facing challenges (Bandura et al., 1999; Qian et al., 2022). The core contribution of this pathway lies in providing clear scaffolds for competence development, transforming external support into learners' internalized, transferable “I can do this” beliefs, and laying the foundation of ability and confidence for sustained engagement.
(2) Contextualized emotional and intercultural care pathway critically satisfies students' relatedness and autonomy needs (Ryan and Deci, 2020). Practices described by T2, T4, and T7 show that by discussing potential cultural misunderstandings in advance, emphasizing communicative intent, and providing emotional acceptance and accompaniment during student anxiety, teachers cultivate a psychologically safe learning environment. Narratives from S3 and S7 clearly indicated that this environment transforms the “fear of being judged” into a “risk worth taking.” This psychological safety is a prerequisite for encouraging students to engage in autonomous exploration and take communicative risks. From an ESD perspective, such care not only alleviates immediate anxiety but also cultivates learners' essential emotional regulation and psychological resilience in intercultural interactions (Kioupi and Voulvoulis, 2019), which are themselves key sustainable development competences. Therefore, the dual-pathway teacher support does not operate in isolation. Structured empowerment enhances students' ability to meet challenges (self-efficacy), while emotional care provides a safe space to exercise that ability (psychological safety). Together, they shift bilingual learning from a “task-performance orientation” toward the cultivation of “communicative competence and psychological capital,” exemplifying the ESD principle of promoting holistic and sustainable learner development (UNESCO, 2020).

### 5.2 Collaborative effects and social dynamics of peer support

Unlike the top-down support provided by teachers, peer support in this study manifested as a horizontal “collaborative effect”, operating through dual modes of cognitive collaboration and emotional resonance, serving as a key social resource that maintains the vitality of the learning community and supports students' psychological wellbeing. This directly addresses RQ2.

(1) Cognitive dimension: peers function as “cognitive collaborators”, offering diverse problem-solving perspectives and constructive feedback. As S1 and S6 described, peer feedback emphasizes “thinking together on how to optimize” rather than simple judgment. This collaborative process fosters co-construction of meaning (Peralta Hernández and Tirado Segura, 2023), exposing students to linguistic resources and strategies beyond individual limitations and continuously inspiring new language use. Such cognitive interaction constitutes an important social driver for knowledge circulation and competence development within the learning ecosystem.
(2) Emotional dimension: peers act as “emotional resonators,” providing irreplaceable affective support. S9 and S6 described the feeling of “advancing together” and shared experiences of facing pressure, exchanging anxieties, and celebrating progress. These experiences effectively alleviate the inherent loneliness and uncertainty stress in bilingual learning (Green et al., 2022). This emotionally grounded connection fosters a strong sense of collective belonging, integrating individual learners' emotional experiences into a supportive social network. This not only enhances students' psychological wellbeing during the learning process but also, by regulating negative emotions and reinforcing positive affective memories, serves as a critical emotional bond that sustains persistence in the long and challenging learning journey. Thus, the “collaborative effect” of peer support demonstrates that peers function not only as sources of information and strategies but also as social conditions for emotional support and motivational maintenance. Cognitively, they expand the boundaries of learning; emotionally, they consolidate learning resilience. Together with teacher support, they form a complementary and indispensable social support network within the sustainable bilingual learning ecosystem.

### 5.3 Internalization of external support: from psychological safety to flow experience

One of the most significant findings of this study is how external social support is internalized by learners and transformed into sustained engagement through psychological processes. This internalization pattern constitutes a continuum from foundational psychological conditions to higher-order experiences, systematically addressing RQ3 regarding the interaction between support systems and intrinsic psychological processes. This continuum is grounded in participants' experiential accounts, which consistently highlight how social support is perceived and internalized in everyday bilingual interactions. Building on these empirically derived insights, the subsequent interpretation draws on relevant theoretical frameworks to further explain the underlying psychological mechanisms.

(1) Internalization into Cross-cultural psychological safety: care from teachers and emotional resonance from peers are jointly internalized as cross-cultural psychological safety, forming the foundation of the internalization process. As S3 and S7 described, when mistakes are regarded as part of the learning process, fear is transformed into manageable risk. This internalized safety represents a deep-level fulfillment of the relatedness need in self-determination theory, alleviating the psychological burden of autonomous or risk-taking communication and providing a prerequisite for the enhancement of interest and exploratory behaviors (Ryan and Deci, 2020).
(2) Reinforcing interest-self-efficacy cycle: on the basis of psychological safety, interest-driven exploration, combined with external recognition from teachers and peers, strengthens the interest-self-efficacy reinforcing loop. S8's narrative illustrates this cycle: interest drives exploration → exploration leads to successful experiences and external recognition → success enhances self-efficacy → increased self-efficacy further fuels interest. This finding confirms the theoretical notion of mutually reinforcing motivation and self-efficacy (Street et al., 2022). This cycle is not a one-time occurrence but functions as a self-reinforcing intrinsic motivational engine through repeated success experiences, serving as a core psychological mechanism for the development of sustainable bilingual competence.
(3) Flow experience in bilingual communication: when external support is fully internalized (sufficient psychological safety) and the intrinsic motivational cycle (interest-self-efficacy) operates efficiently, some learners experience flow during bilingual interaction. S3 and S9 described a state of high immersion in which they “forgot which language they were using”, emerging from the combination of challenge-skill balance, autonomous control, and clear goals. This flow experience has a dual reinforcing effect: (1) it enhances the quality and enjoyment of individual learning episodes; (2) as a positive experiential memory, it fosters emotional attachment to bilingual practice and investment in identity, making learners “eager to engage again”, thereby strongly promoting long-term, voluntary, and sustained participation.

### 5.4 Theoretical contributions

Based on the above analysis, the theoretical contributions of this study can be summarized in three main aspects:

(1) By presenting the “dual-pathway model” of teacher support (structured empowerment and contextualized care), this study deepens the understanding of the mechanisms through which teacher support operates within a sustainable learning ecosystem. Specifically, effective teacher support must simultaneously attend to the development of both students' competence systems and psychological support systems, providing detailed theoretical guidance for designing instructional practices that support long-term student development within the ESD framework.
(2) By conceptualizing peer support as a *cognitive collaboration* and *emotional resonance* collaborative effect, this study clarifies that peer relationships serve not only as cognitive resources but also as key emotional and motivational maintenance systems. This addresses the potential overemphasis on teacher roles in existing research and elevates peer support to an equally important and independently valuable social pillar within the sustainable learning ecosystem.
(3) This study integrates and proposes an internalization model: psychological safety → interest-self-efficacy reinforcing cycle → flow experience and identity investment. This model clearly demonstrates how external social support, through satisfying basic psychological needs and enhancing intrinsic motivational cycles, translates into high-quality learning experiences and long-term identity commitment. It provides a coherent psychological mechanism framework for understanding the development and maintenance of sustainable bilingual competence, connecting social support theory, self-determination theory, and social cognitive theory.

### 5.5 Research limitations and future directions

Despite the in-depth qualitative analysis revealing the key mechanisms of the sustainable bilingual learning ecosystem, some limitations should be acknowledged: (1) The cross-sectional qualitative design, while enabling detailed observation of mechanisms and experiences, limits the ability to dynamically track the longitudinal evolution of individual psychological processes, such as self-efficacy and identity development; (2) Participants were drawn from specific disciplines within Korean higher education, resulting in a relatively homogeneous cultural and educational background, which may constrain the generalizability of the model across different contexts. Accordingly, the findings should be interpreted in terms of analytical transferability rather than statistical generalizability. The model is grounded in a specific higher education context and is most applicable to similar bilingual or multilingual settings, while contextual factors (e.g., institutional structures, language configurations, and curricula) may influence its applicability; (3) Data primarily relied on self-reported interviews, without incorporating classroom observations, learning logs, or other triangulated data sources, which may limit methodological robustness; and (4) The study focused on micro-level classroom interactions, without examining how macro-level structures such as institutional policies or curriculum frameworks influence the learning ecosystem.

To address these limitations, future research could: (1) employ longitudinal tracking or diary-based designs to dynamically depict the internalization of support and psychological development processes; (2) Test and refine the model across different countries, educational levels, and disciplinary contexts in bilingual/multilingual classrooms to enhance cross-context applicability; (3) Incorporate classroom video observations, learning behavior data, or standardized instruments in mixed-methods studies to improve the robustness and ecological validity of findings; and (4) Conduct multi-level analyses to explore how macro-level institutional factors interact with micro-level classroom interactions in shaping the structure and effectiveness of sustainable learning ecosystems.

## 6 Conclusion

Informed by the ESD perspective, this study employed constructivist grounded theory and a three-level coding procedure to systematically analyze the interview transcripts of teachers and students. The findings indicate that the development of sustainable bilingual competence is not incidental, but rather emerges from the interaction between external social support and the learners' internal psychological structures. These conclusions are grounded in participants' reported experiences of sustained engagement, perceived psychological safety, and evolving bilingual competence within their learning contexts, rather than being derived solely from normative sustainability frameworks. The core conclusions can be integrated and articulated within the proposed sustainable bilingual learning ecosystem model as follows: (1) through the dual-pathway model of structured language empowerment and situated emotional and cross-cultural care, teachers achieve a balance between enhancing linguistic communicative skills and ensuring psychological safety, thereby laying the foundational conditions for long-term bilingual engagement. Additionally, peers contribute through the synergistic effects of cognitive collaboration and emotional resonance, constructing a learning community supported by multiple perspectives and emotional connections, which serves as a central social resource for maintaining learning motivation and resilience; and (2) The study also traced the internal psychological pathways, beginning with the cross-cultural psychological safety internalized from environmental support, which subsequently strengthens the interest and enhance self-efficacy loop. When conditions are met, leads to bilingual flow experiences and identity investment. This indicates that, at the psychological level, sustainable bilingual competence constitutes a self-sustaining, self-reinforcing motivational and developmental process. Consequently, the framework constructed in this study reconceptualizes the sustainability of bilingual learning as a co-constructed ecosystem of external support and internal psychological processes. This model provides an integrative perspective for understanding the long-term development, sustained engagement, and identity transformation in higher education bilingual competence, while also offering theoretical guidance and practical implications for implementing ESD principles and designing bilingual teaching practices that foster a sustainable learning ecosystem.

## Data Availability

The original contributions presented in the study are included in the article/Supplementary material, further inquiries can be directed to the corresponding author.
