# Supplementary material for: A sustainable bilingual learning ecosystem in higher education: teacher and peer support under SDG 4.7
Source: Front Psychol. 2026 May 14;17:1809800. doi: 10.3389/fpsyg.2026.1809800 (PMC13216474; doi:10.3389/fpsyg.2026.1809800)
Supplement: Supplementary file 1 [file Supplementary_file_1.docx]

**Supplementary Table S1. Sampling Framework and Participant Characteristics**

| **Participant Group** | **Sampling Dimension** | **Inclusion Criteria** | **N** | **Key Characteristics** |
| --- | --- | --- | --- | --- |
| Teachers (N = 10) | Academic field & teaching context | Faculty members from English-related departments (e.g., English Language and Literature, Applied Linguistics, TESOL), teaching Chinese as a Second Language (CSL) within bilingual programs at two comprehensive universities in Gyeongsangbuk-do, South Korea | 10 | Teaching experience: 5–12 years; instructional contexts include academic Chinese writing, oral communication, and intercultural tasks |
|  | Teaching experience | Mid-career (5–8 years) and senior (9–12 years) teachers | 5 + 5 | Ensures representation of both innovative practices and accumulated pedagogical expertise |
|  | Support orientation | Identified via pre-interview screening as differing in emphasis on structured linguistic scaffolding, emotional care, and intercultural support | – | Captures the full spectrum of teacher support practices relevant to sustainable bilingual learning |
| Students (N = 10) | Academic major & language background | Undergraduate students majoring in English-related programs; officially enrolled in intermediate-to-advanced Chinese courses | 10 | Year 2–4 undergraduates; Chinese proficiency approximately HSK 3–5 or equivalent |
|  | Perceived support & motivation | Screened to ensure variation in perceived teacher support, peer support, and self-reported sustained motivation for Chinese learning | – | Ensures heterogeneity in support experiences and motivational trajectories |
|  | Learning engagement patterns | Sampled based on differences in behavioral, cognitive, and emotional engagement across academic and social bilingual contexts | – | Enables comparison of how support mechanisms operate under different engagement profiles |

**Supplementary Table S2. Core Questions in the Semi-Structured Interview Protocol**

| **Participant Group** | **Core Construct** | **Sample Questions** |
| --- | --- | --- |
| Teachers | Teacher support | 1. What instructional strategies do you find most effective in sustaining students’ long-term interest and confidence in using Chinese, both inside and outside the classroom? Please provide concrete examples.   2. When students experience anxiety or low self-efficacy in cross-linguistic tasks (e.g., English–Chinese academic writing or presentations), how do you typically intervene, and why? |
|  | Peer support | 3. How do you design or facilitate learning activities that promote meaningful peer interaction in bilingual (English–Chinese) contexts? What learning or psychological outcomes have you observed? |
|  | Sustainable bilingual learning ecosystem | 4. In your view, what characterizes a “sustainable bilingual learning ecosystem” that supports long-term language development and intercultural competence? How do you attempt to cultivate such an environment in your teaching? |
| Students | Teacher support | 1. Please describe a specific instance in which a teacher’s support significantly increased your willingness or confidence to use Chinese. What exactly did the teacher do or say?2. What types of feedback on your Chinese use (accuracy, fluency, pragmatics) have been most helpful for sustaining your learning motivation, and why? |
|  | Peer support | 3. What role do peers play in your bilingual (English–Chinese) learning journey? Please share an example in which peer interaction positively influenced your language use or persistence. |
|  | Motivation, self-efficacy & sustained engagement | 1. When do you feel most engaged and effective in bilingual tasks? What factors contribute to this state, and how do interest and self-efficacy interact in your experience?   5. Despite challenges, what motivates you to persist in learning Chinese over time? What social or environmental factors support this persistence? |
|  | Sustainable bilingual learning ecosystem | 6. How would you describe a learning environment that continuously supports your growth as a bilingual user? Which classroom or community experiences make you feel that your bilingual competence is genuinely developing and valued? |

**Supplementary Table S3. Three-Level Coding Procedures Based on Constructivist Grounded Theory**

| **Coding Stage** | **Analytic Focus** | **Procedures and Examples** | **Outputs** |
| --- | --- | --- | --- |
| Open Coding | Conceptualizing raw data | Line-by-line analysis of interview transcripts to identify initial concepts related to teacher support, peer interaction, learning motivation, L2 self-efficacy, emotional experiences, and sustained engagement.Examples:(1) “Differentiated linguistic scaffolding” (teachers provide tailored feedback on grammar and pragmatics); and (2) “Peer modeling reduces affective filter” (observing peers’ successful communication lowers anxiety). | 118 initial codes |
| Axial Coding | Establishing relationships among concepts | Grouping and integrating initial codes based on shared properties and relational patterns.Examples:(1) “Differentiated scaffolding” + “Strategic instructional guidance” → Structured linguistic empowerment; and (2) “Anxiety acknowledgment” + “Autonomy in topic selection” → Contextualized emotional and motivational support. | Higher-order categories |
| Selective Coding | Integrating categories into a core theme | Identifying “Sustainable Bilingual Learning Ecosystem” as the core category, integrating teacher support, peer support, and internal psychological processes (psychological safety, motivation, self-efficacy) into an explanatory ecosystem model. | Core theme and theoretical model |
| Trustworthiness Strategies | Ensuring rigor | (1) Inter-coder agreement: Two researchers independently coded 30% of transcripts (Cohen’s κ = 0.76); (2) Member checking with selected participants; (3) Theoretical saturation confirmed after the 8th teacher and 9th student interviews; and (4) Ongoing reflexive memo writing. | Credibility and transparency ensured |

**Supplementary Table S4. Theme One: Dual-Pathway Model of Teacher Support in Bilingual Learning**

| **Main Category** | **Subcategory** | **Representative Quotes (Teachers)** | **Representative Quotes (Students)** |
| --- | --- | --- | --- |
| Structured Linguistic Empowerment | Competence-oriented strategies | “When teaching academic Chinese writing, I first deconstruct rhetorical structures, then guide students to identify problems in sample texts, and finally provide targeted feedback for their own drafts.” (T4, T1, T7) | “My vocabulary was fine, but cohesion was weak. The teacher compared English and Chinese editorials. This wasn’t just error correction—it helped me develop a more mature writing style.” (S5, S2, S8) |
|  | Autonomy-supportive strategies | “Although core language objectives are fixed, students choose their own presentation topics—K-pop, Sino-Korean relations, or economics. Autonomy drives deeper engagement.” (T3, T5, T9) | “My advisor encouraged me to compare English and Chinese medical texts based on my research interest. That autonomy made language learning personally meaningful and sustainable.” (S10, S3, S6) |
| Contextualized Emotional and Intercultural Care | Emotional support strategies | “A student froze during a Chinese business simulation. Instead of drilling sentences, we discussed cultural concepts of ‘face’ across languages. Understanding the source of anxiety reduced performance pressure.” (T6, T2, T8, T10) | “I feared using idioms. My teacher said, ‘One awkward but sincere attempt is better than ten perfect silences.’ That changed my mindset from avoiding mistakes to valuing attempts.” (S1, S4, S7, S9) |

**Supplementary Table S5. Theme Two: Collaborative Effects of Peer Support in Bilingual Learning**

| **Main Category** | **Subcategory** | **Representative Quotes (Students)** |
| --- | --- | --- |
| Cognitive Collaboration | Multi-perspective feedback | “In peer review, a linguistics major pointed out that my Chinese sentences followed English logic. That insight helped me rethink how to construct arguments in Chinese.” (S4) |
|  | Vicarious learning and modeling | “Seeing a peer clearly explain a complex idea in Chinese during a seminar gave me a concrete model. I thought, ‘If she can do it that way, I can try too.’” (S7) |
| Emotional Resonance | Shared bilingual experiences | “When preparing for HSK 5, our study group became a safe space. We shared frustrations, celebrated small wins, and naturally switched languages. That sense of ‘we’re in this together’ kept us going.” (S9) |
|  | Constructive peer feedback | “Good peer feedback sounds like: ‘This part is clear, but maybe this phrase could make it smoother.’ It feels like collaborative problem-solving, not judgment.” (S3, S6) |

**Supplementary Table S6. Theme Three: Internalization of Support and Transformation toward Sustainable Bilingual Engagement**

| **Psychological Stage** | **Qualitative Evidence (Illustrative Patterns)** |
| --- | --- |
| Development of Cross-Cultural Psychological Safety | Students consistently emphasized that teachers’ emotional care and peers’ shared struggles created the foundation for “daring to use the language” in authentic, high-risk contexts. Without this safety, deep linguistic and intercultural experimentation was inhibited. |
| Interest–Self-Efficacy Reinforcing Cycle | Participants described a reinforcing loop: initial interest in Chinese culture → active language exploration → successful communicative experiences → enhanced L2 self-efficacy → deepened motivation and engagement  (e.g., consuming authentic media, forming friendships). |
| Bilingual Flow Experience and Sustainable Identity Investment | When external support satisfied psychological needs, students reported immersive “flow” states in bilingual interaction. These positive experiences strengthened their identity as competent bilingual users and motivated long-term investment in maintaining and developing bilingual competence. |
